# The value of early and comprehensive diagnoses in a human fetus with hydrocephalus and progressive obliteration of the aqueduct of Sylvius: Case Report

**DOI:** 10.1186/s12883-016-0566-7

**Published:** 2016-04-11

**Authors:** Eduardo Ortega, Rosa I. Muñoz, Nelly Luza, Francisco Guerra, Monserrat Guerra, Karin Vio, Roberto Henzi, Jaime Jaque, Sara Rodriguez, James P. McAllister, Esteban Rodriguez

**Affiliations:** Unidad de Neurocirugía, Instituto de Neurociencias Clínicas, Facultad de Medicina, Universidad Austral de Chile, Valdivia, Chile; Instituto de Anatomía, Histología y Patología, Facultad de Medicina, Universidad Austral de Chile, Valdivia, Chile; Instituto de Fisiología, Facultad de Medicina, Universidad Austral de Chile, Casilla 456, Valdivia, Chile; Department of Neurosurgery, Division of Pediatric Neurosurgery, Washington University School of Medicine, St. Louis, Missouri USA

**Keywords:** Congenital hydrocephalus, Aqueduct of Sylvius, Cerebral aqueduct, Stenosis, Subcommissural organ, Cerebrospinal fluid, Case study

## Abstract

**Background:**

Mutant rodent models have highlighted the importance of the ventricular ependymal cells and the subcommissural organ (a brain gland secreting glycoproteins into the cerebrospinal fluid) in the development of fetal onset hydrocephalus. Evidence indicates that communicating and non-communicating hydrocephalus can be two sequential phases of a single pathological phenomenon triggered by ependymal disruption and/or abnormal function of the subcommissural organ. We have hypothesized that a similar phenomenon may occur in human cases with fetal onset hydrocephalus.

**Case presentation:**

We report here on a case of human fetal communicating hydrocephalus with no central nervous system abnormalities other than stenosis of the aqueduct of Sylvius (SA) that became non-communicating hydrocephalus during the first postnatal week due to obliteration of the cerebral aqueduct. The case was followed closely by a team of basic and clinic investigators allowing an early diagnosis and prediction of the evolving pathophysiology. This information prompted neurosurgeons to perform a third ventriculostomy at postnatal day 14. The fetus was monitored by ultrasound, computerized axial tomography and magnetic resonance imaging (MRI). After birth, the follow up was by MRI, electroencephalography and neurological and neurocognitive assessments. Cerebrospinal fluid (CSF) collected at surgery showed abnormalities in the subcommissural organ proteins and the membrane proteins L1-neural cell adhesion molecule and aquaporin-4. The neurological and neurocognitive assessments at 3 and 6 years of age showed neurological impairments (epilepsy and cognitive deficits).

**Conclusions:**

(1) In a hydrocephalic fetus, a stenosed SA can become obliterated at perinatal stages. (2) In the case reported, a close follow up of a communicating hydrocephalus detected *in utero* allowed a prompt postnatal surgery aiming to avoid as much brain damage as possible. (3) The clinical and pathological evolution of this patient supports the possibility that the progressive stenosis of the SA initiated during the embryonic period may have resulted from ependymal disruption of the cerebral aqueduct and dysfunction of the subcommissural organ. The analysis of subcommissural organ glycoproteins present in the CSF may be a valuable diagnostic tool for the pathogenesis of congenital hydrocephalus.

## Background

Hydrocephalus is a condition in which cerebrospinal fluid (CSF) accumulates within the brain ventricles, resulting in ventricular dilatation and often increased intracranial pressure [1]. It is now understood that hydrocephalus is not only a disorder of CSF dynamics, but also a brain disorder. The mechanistic views of brain damage caused by increased intracranial pressure do not answer the inborn and irreparable neurological impairment of children with fetal onset hydrocephalus [2–4]. Recent studies in mutant animals [5–7] and in human hydrocephalic fetuses [8–12] have shown that the loss of neural stem cells (NSC)/ependymal cells forming the ventricular zone (VZ) during embryonic development is associated with both hydrocephalus and abnormal neurogenesis. The ventricular zone disruption leading to hydrocephalus and abnormal neurogenesis may result from gene mutations or environmental factors, such as intraventricular hemorrhage or folic acid deficiency [11]. Many gene mutations underlying VZ disruption have been reported, all of them involved in intracellular trafficking [12]. In addition, pathology of the subcommissural organ (SCO), an ancient gland secreting glycoproteins into the CSF, has been associated with the stenosis/obliteration of the aqueduct of Sylvius (SA) and fetal onset hydrocephalus [13–19].

Here we describe the clinical and pathological evolution of a patient that was diagnosed *in utero* with a progressive obliteration of the SA and hydrocephalus and with abnormalities in SCO secretory proteins. The case was followed by a multidisciplinary team that prompted neurosurgeons to perform an early endoscopic third ventriculostomy. The evidence collected indicates that such a surgery did not resolve abnormalities in neurogenesis but completely prevented secondary brain damage. This case illustrates the importance of: (1) making an early diagnosis of fetal hydrocephalus and closely following its evolution; (2) being aware of the probable mechanisms underlying the transition from a mild communicating hydrocephalus to a severe non-communicating hydrocephalus; and (3) being conscious of the cellular mechanisms underlying the long-term neurological outcomes.

## Case presentation

### Clinical findings

**Fig. 1:**
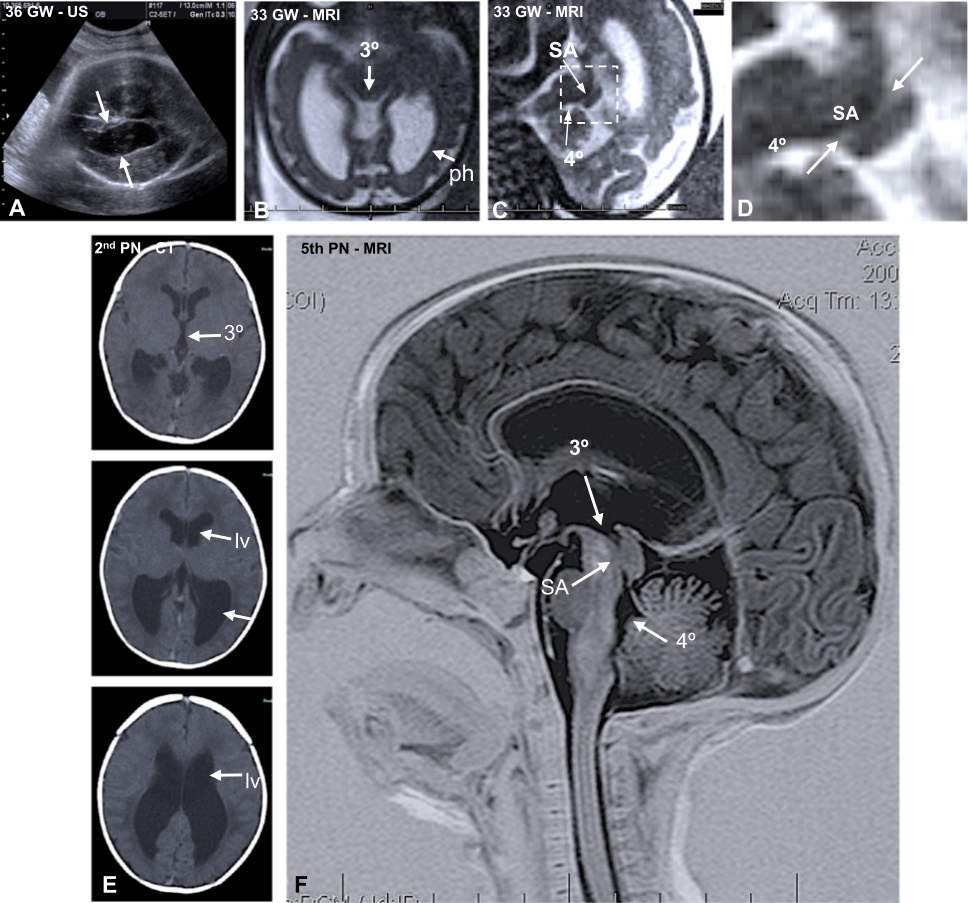
Progressive obliteration of the aqueduct of Sylvius (SA) in hydrocephalus. **a** An ultrasound of the fetal patient at 36 GW demonstrating dilation of the lateral ventricles (arrows). **b** MRI at 33 GW. The third ventricle (3°) and posterior horns of the lateral ventricles (ph) are dilated. **c** MRI at 33 weeks. Stenosis of the SA is shown. **d** Detailed magnification of the area framed in C showing stenosis of the SA. 4°, fourth ventricle. **e** CT at 39 GW (or the 2nd PN day). The lateral (LV) and third (3°) ventricles are dilated. **f** MRI of the brain on the 5th postnatal day, sagittal T2 imaging. The SA is obliterated

An ultrasound (US) examination was performed at 28 gestational weeks (GW) on a woman aged 39 who had 3 previous pregnancies. Detailed two-dimensional (2D) and three-dimensional (3D) US examinations were performed with a scanner (Medison Model 8000) and a trans-abdominal transducer (2–5 MHz). It revealed a female fetus with biometry consistent with 28 GW. A posterior US confirmed the ventriculomegaly. Brain fetal biometry parameters were as follows: biparietal diameter 7.51 mm, head circumference 295.6 mm, posterior horn of the lateral ventricle width 19.3 mm. These parameters are in the upper 90th percentile for gestational age and are suggestive of ventriculomegaly. No other abnormal findings were obtained. Figure 1a shows the US performed at 36 GW, with a lateral ventricle width of 22.3 mm. A follow-up magnetic resonance imaging (MRI) examination performed at 33 GW revealed stenosis of the SA and ventriculomegaly, confirming hydrocephalus (Fig. 1b-d). At 37 GW, an elective C-section was performed and a baby girl was delivered with a weight of 2,750 g, a length of 45 cm (which is below the 10th percentile for gestational age), a cranial circumference of 36.5 cm > P 98, and Apgar scores of 9 and 9 after 1 and 5 min, respectively. An examination of the neonate showed a normal external morphology.

At postnatal day 2 a computerized axial tomography study confirmed the large expansion of the third and lateral ventricles (Fig. 1e). The final diagnosis was fetal onset hydrocephalus. MRI performed on the 5th postnatal day showed SA obliteration (Fig. 1f). An endoscopic third ventriculostomy was performed on the 14th postnatal day and ventricular CSF was collected. The penetration site of neuroendoscope was the prefrontal region of the right hemisphere. Direct endoscopic visualization from the third ventricle showed complete occlusion of the SA. Since in animal models the subcommissural organ (SCO) is involved in the SA stenosis/obliteration [13–19], CSF samples were processed to study SCO secretory proteins (see below). In the immediate postoperative period the patient developed ventriculitis and fever prompting external ventricular drainage for ten days and treatment with Ceftriaxone (100 mg/Kg/day) and Vancomycine IV (10 mg/Kg/day). Following a good response to antibiotic treatment the patient left the Pediatrics Intensive Care Unit on the 60th postnatal day.

During the patient’s postnatal course, MRI and electroencephalography (EEG) studies, cognitive tests and CSF analyses were performed. Parental informed consent and approval from the Ethics Committees of the Universidad Austral de Chile, Valdivia, Chile and the Hospital Regional Valdivia, Chile, were obtained.

**Fig. 2:**
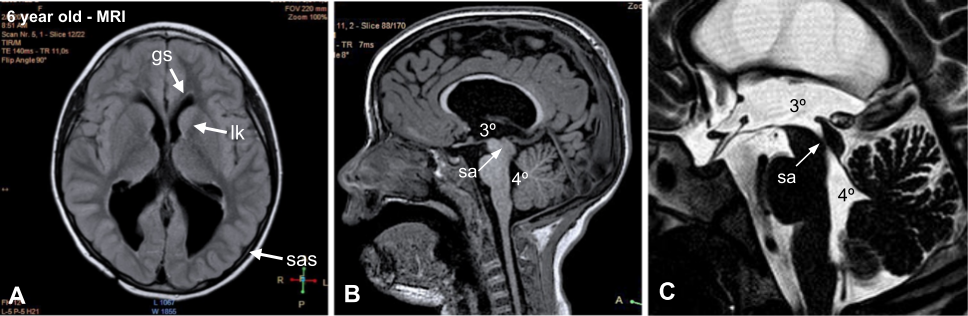
MRI findings at 6th years of age. **a** MRI (transverse T1 imaging) showing mild ventricular dilatation, a normal subarachnoid space (sas), signs of periventricular leukomalacia (lk) in the frontal horns of the lateral ventricles, and a glial scar (gs) in the frontal subcortical zone. **b**, **c** MRI (sagittal T1 and sagittal T2 imaging, respectively) showing complete obliteration of the SA

A MRI performed at 6 years of age showed obliteration of the SA, mild dilatation of the lateral ventricles, a normal subarachnoid space, signs of periventricular leukomalacia in the frontal horns of the lateral ventricles, and a zone suggesting a glial scar in the frontal subcortical zone (Fig. 2).

At 14 months of age the patient had an initial generalized tonic-clonic seizure while she was sleeping (1:00–2:00 am). Phenobarbital (6 mg/kg per day) was used as monotherapy to treat the seizures with an adequate response. Subsequently, the patient had four additional tonic-clonic seizures during sleep, all of them associated with a decrease of the Phenobarbital dose. The EEG study showed epileptiform specific activity in the posterior frontal region with rapid intercritic generalization.

### Psychomotor development

Her psychomotor development included sitting without support during the 7th month, first intentional spoken words during the 8th month, walking beginning during the 17th month, and sphincter control by the 19th month. At the 21st month the psychomotor development was evaluated as normal by using the Psychomotor Development Test EEDP [20].

### Neurocognitive assessment

The patient was examined by a clinical neuropsychologist at 3 and 6 years of age. During the 3rd year, a TEPSI test aimed at the assessment of coordination, language and motor development [20] was normal. However, a discrepancy between the scales was evident; she excelled in language but was below normal in coordination and motor development. During the 6th year, a comprehensive neuropsychological test battery with a structured interview was performed for several cognitive functions aimed at the assessment of global cognitive functioning, memory, language, processing speed, visual-spatial abilities, and executive functions. The following tests were used: Wechsler Intelligence Scale for Children (WISC-R) [21, 22], Bender Visual Motor Gestalt Test (BVMG) [23, 24], and Goodenough Test [25, 26]. Each valuation was performed in individual sessions.

According to the results of performance subtests (picture completion, block design, matrix reasoning and coding) of the WISC-R, the Intelligence Quotient (IQ) score was normal (97). The patient had good verbal conceptualization and language development accompanied by an appropriate numerical reasoning to age. She excelled on the Verbal Scale (112) but the Verbal Executive scale was below the normal range (80); this discrepancy was statistically significant (*p* >0.95), suggesting the existence of a neurological disorder. In addition, the patient showed poor hand-eye coordination and an overall decreased maturation level, ranging from 5.0 to 5.1 years old. She showed an appropriate long-term memory but diminished working and short-term memories, with serious interference by hyperactivity, abnormal attention, and concentration skills. She also had difficulties in abstract thinking, synthesis and categorization. Concerning social abilities, the patient had a very good deductive reasoning and an adequate planning capacity, contrasting with her diminished ability to cope and manage problems and situations of everyday life.

### Molecular analysis

**Fig. 3:**
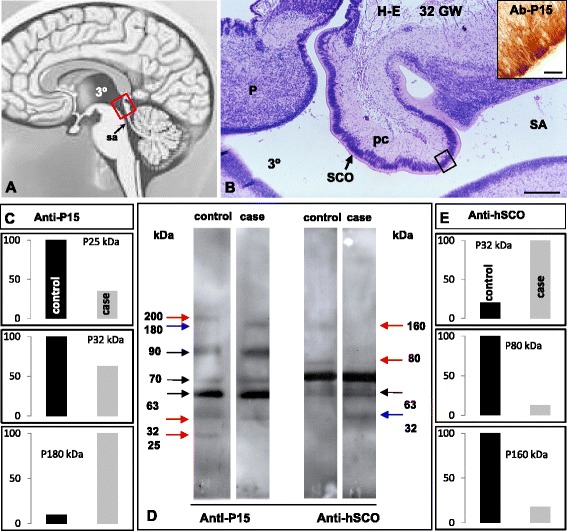
The human subcommissural organ (SCO). **a** Line drawing of a human brain showing the location of the SCO (red rectangle). **b** Sagittal section through the epithalamus of a 32 GW human embryo (for orientation see rectangle in previous figure) showing the SCO, posterior commissure (pm), pineal gland (P), third ventricle (3°) and SA. Inset. Ependyma of a human SCO (see frame in B), immunostained for SCO-spondin. Scale bar B 350 µm, inset 50 µm. **c** Histograms of microdensitometric recordings of immunoblots with anti-P15, shown in (**d**). **d** Immunoblots of CSF samples from the hydrocephalic case and from a 33rd GW fetus with an arachnoid cyst, using anti-P15 and anti-hSCO antisera. Blue arrows point to compounds present in the hydrocephalic CSF and missing in the control; red arrows indicate compounds that are present in the control but missing or at lower concentration in the hydrocephalic CSF. **e** Histograms of microdensitometric recording of immunoblots with anti-P15, shown in (**d**). Control, CSF obtained by lumbar puncture from a patient 9 months old diagnosed with leukemia symptoms but no ventriculomegaly

The subcommissural organ is phylogenetically the most ancient brain gland, developing early in ontogeny [27, 28]. In the human it is fully developed by the 12th GW [28]. It is located in the roof of the third ventricle at the entrance of the SA (Fig. 3a, b). The SCO secretes into the CSF two classes of proteins: the ones that remain soluble in the CSF and consequently move with the flow of CSF and those that aggregate to form an insoluble ever-growing structure, the Reissner fiber [27–29]. SCO-spondin is the main protein secreted by the SCO [30] and its complete sequence was reported by Gobron et al. (2000) [31]. Proteins of 200, 63, 50, and 25 kDa reacting with antibodies specific for SCO-spondin have been consistently found in the CSF of normal rodents. These proteins most likely result from processing of SCO-spondin [32].

**Fig. 4:**
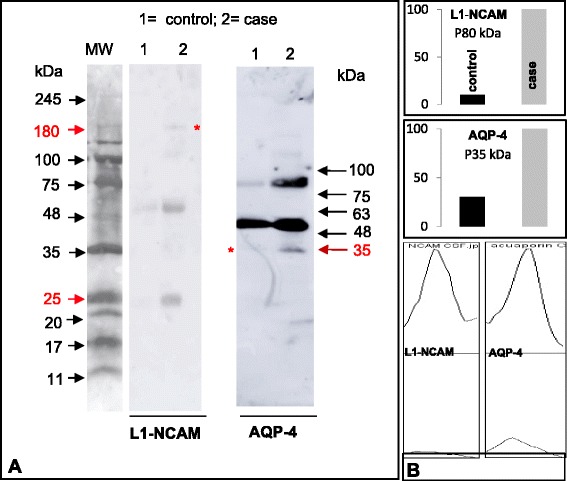
Proteins of ependymal cells are present in the hydrocephalic CSF. **a** Immunoblots of CSF samples from the hydrocephalic case and a control using antibodies against L1-CAM) and AQP-4. The 180 kDa form of L1-CAM (red arrow and star) and other compounds reacting with anti- L1-CAM are detectable in the hydrocephalic CSF but not in the control. The 35 kDa form of AQP-4 (red arrow and star) is readily detectable in the hydrocephalic CSF but not in the control. **b** Histograms of microdensitometric recordings of immunoblots with anti- L1-CAM and anti-AQP-4, shown in (**a**). Control, CSF obtained from a fetus with an arachnoid cyst, at 33rd GW

In the present case, immunoblot analyses of CSF collected at the time of endoscopic third ventriculostomy were performed using antibodies against: (1) P15, a specific peptide whose sequence is unique for SCO-spondin [28]; (2) a protein extract of human subcommissural organ (anti-hSCO) [28]; (3) L1-CAM, an adhesion molecule that has been found in the CSF of hydrocephalic newborns [33] and; (4) aquaporin-4 (anti-AQP-4), a water channel selectively expressed by ependymal cells and astrocytes [34–36]. Anti-P15 reacted with cells of the SCO (Fig. 3b, inset). The pattern of anti-P15 reactive compounds in the control CSF resembles that of the CSF of normal rats [32]. The CSF in the present case showed a pattern of anti-P15 reactive compounds different from that of historical controls; the compounds of 200 and 25 kDa were missing and an abnormal form of 180 kDa was present (Fig. 3c, d). The anti-hSCO also revealed differences between the CSF of the hydrocephalic patient and that of control (Fig. 3e). The L1-CAM and anti-AQP-4 were barely distinguished in the control CSF but distinctly detected in the CSF of the hydrocephalic patient (Fig. 4a, b).

## Discussion

### A Flecha chart summarizing key events of case report is shown in the Fig. 5

On the basis of previous experimental evidence, the evolution of the patient reported was closely followed by a team of basic and clinic investigators, allowing an early diagnosis and confirmation of a predicted clinical and pathophysiological progression. In this case, an early postnatal endoscopic third ventriculostomy was performed and at six years of age the overall outcome of this child showed only a minor neurological impairment. The evolution of a communicating to a non-communicating hydrocephalus should be considered when SA stenosis is diagnosed *in utero*.

**Fig. 5:**
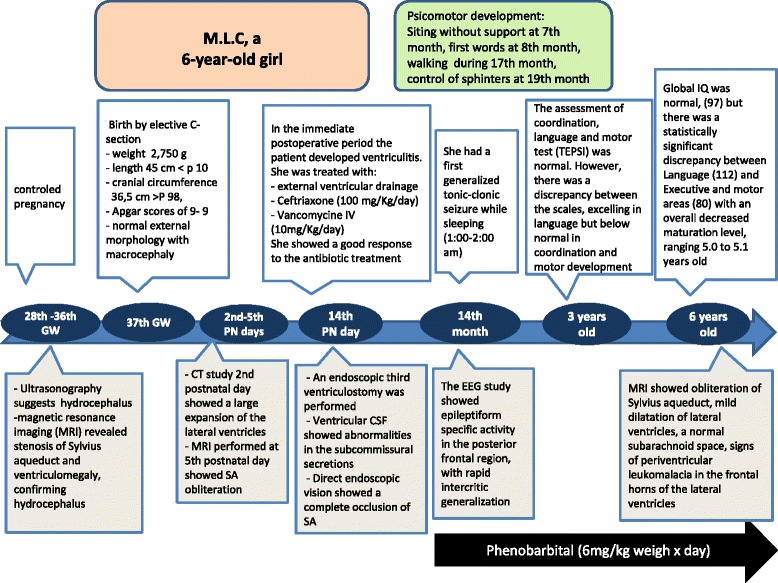
Flecha chart of case report. Key events are summarized

The clinical and pathological (imaging, neurocognitive assessment, CSF analyses) evolution of the present patient with fetal onset hydrocephalus supports the possibility that the progressive stenosis of SA initiated during the embryonic period may have resulted from (1) ependymal disruption of the cerebral aqueduct and/or (2) dysfunction of the subcommissural organ.

A strong body of evidence obtained in mutant hydrocephalic animals and human hydrocephalus indicates that the loss of neural stem cells/ependyma from the embryonic germinal zone, regarded as ventricular zone disruption, is associated with the onset of hydrocephalus [5–12]. The disruption phenomenon is the final outcome of several genetic and non-genetic abnormalities that lead to the abnormal expression of gap and adherent junctions [11, 12, 37–39]. In mutant *hyh* mice, the time program of ventricular zone disruption starts during fetal life and ends by the end of the first postnatal week [5–7]. It is only after this disruption program has been completed that the denuded walls of the SA fuse and obliteration of the SA takes place, triggering ventriculomegaly. Similar abnormalities of gap and adherent junctions have been also reported in the SA of full-term hydrocephalic human fetuses [10–12]. In animal models, environmental factors, such as intraventricular hemorrhage or folic acid deficiency, as well as a long series of gene mutations underlying the junction pathology of the ventricular zone preceding hydrocephalus have been reported [1, 11, 12]. Although human hydrocephalic fetuses also display a junctional pathology of the ventricular zone, gene mutations resulting in such a pathology have not yet been identified [8]. In the case reported, no intraventricular hemorrhage was detected, and deficiency of folic acid may be ruled out since in Chile folic acid is included in the diet through the wheat powder. Thus, it seems likely that in this case the progressive SA stenosis, with no other neural tube malformation, is associated with an as yet unknown gene mutation.

The following evidence suggests that disruption of the ependyma of the cerebral aqueduct may have been part of the mechanism leading to SA stenosis found in the present case: (1) hydrocephalus was already detected at the 28th GW, with no other apparent neural tube malformations; (2) stenosis of the SA was detected at the 33rd GW and at the 3rd postnatal day the SA was already obliterated, thus resembling the time sequence found in mutant mice (see above); (3) at six years of age, the SA continued to be obliterated as expected from the lack of regeneration capacity of ependyma; (4) the presence in the CSF, collected on the 14th postnatal day, of proteins present in the plasma membrane of ependymal cells, such as the L1 cell adhesion molecule and aquaporin 4, may reflect ependymal disruption [33–36].

### The role of the subcommissural organ in fetal onset hydrocephalus

A strong body of evidence indicates that the SCO plays a key role in CSF flow and that its abnormal development in mutant animals leads to SA stenosis and hydrocephalus [13–19]. There is evidence that the negatively-charged SCO-spondin plays a key role in keeping the SA open, and that its absence or its abnormal molecular forms trigger SA stenosis [13, 40–42]. The abnormal pattern of SCO-spondin-related polypeptides in the CSF of this case suggests that abnormalities of the SCO were part of the mechanism leading to the progressive stenosis of SA. In the hydrocephalic mutant HTx rat the abnormal development of the SCO sequentially results in the secretion of abnormal forms of SCO-spondin, SA obliteration and hydrocephalus [13]. It seems likely that this could also be the situation in the present human case.

### Hydrocephalus and abnormal neurogenesis as a primary pathology of neural stem cells/ ependymal cells

In mutant hydrocephalic animals and in humans with fetal onset hydrocephalus, the disruption process of the ventricular zone starts in the SA (see above) and spreads to the telencephalon [5–12], where the disruption phenomenon strikes the ventricular zone formed by neural stem cells, resulting in several brain abnormalities. The most consistent abnormalities are: (1) loss of a pool of neural stem cells and progenitor cells due to their abnormal translocation into the CSF; and (2) formation of periventricular heterotopias due to abnormal migration of neuroblasts [12, 39, 43, 44]. Periventricular heterotopias behave as epileptogenic foci [45]. This may explain why 6–30 % of hydrocephalic children, including the present case, develop epilepsy that is not solved by CSF drainage surgery [46–49]. Thus, the epilepsy and the mild cognitive impairment developed by the patient reported here could both be explained by a disruption of the ventricular zone of the telencephalon. It may be proposed that in the present case the disruption of the ependyma of the SA lead to hydrocephalus and a parallel disruption of neural stem cells in the telencephalon results in a neurological impairment. A key distinction has to be made between the inborn *abnormal* neurogenesis/ependymogenesis and brain *damage* caused by hydrocephalus. Surgical treatments cannot resolve the former but they could prevent further damage. Although unlikely, the possibility that the discrete damage to the cerebral cortex caused during third ventriculostomy may have long term outcomes, has to be considered. Indeed, It is well substantiated that endoscopic third ventriculostomy results in a high rate (around 90 %) of good long-term outcome in patients with obstructive hydrocephalus [50–52].

## Conclusions

Three important messages should be remembered from this case report: (1) make an early diagnosis of fetal hydrocephalus and closely follow its evolution; (2) be aware of the probable mechanisms underlying the transition from a mild communicating hydrocephalus to a severe non-communicating hydrocephalus; (3) be conscious of the cellular mechanisms underlying the long-term neurological outcomes.

### Consent

Written informed consent for publication of this case report and accompanying images was obtained from the mother of the patient. A copy of the written consent is available for review by the Editor-in-Chief of this journal.

## Materials and methods

### CSF collection

Case Patient: CSF was collected from a lateral ventricle while endoscopic third ventriculostomy was performed on the 14th postnatal day. Controls: (1) CSF obtained from a fetus with an arachnoid cyst at the 33rd GW. (2) CSF obtained by lumbar puncture from a 9 month old patient diagnosed with leukemia symptoms. CSF samples were centrifuged twice to remove cells or cell debris. Samples were stored at −70 °C until analysis. Parental informed consent and approval from the Ethics Committee of the Universidad Austral de Chile and Hospital Regional Valdivia, Chile, were obtained.

### Immunoblotting

Sodium dodecyl sulfate polyacrylamide gel electrophoresis (SDS-PAGE) was performed according to the Laemmli method [53]. Briefly, 15 μl undiluted/non-concentrated CSF samples were subjected to SDS-PAGE by using a 5 %–15 % polyacrylamide linear gradient. Proteins were transferred to nitrocellulose membranes [54]. To block nonspecific binding, blots were saturated with 5 % non-fat milk in 0.1 M phosphate-buffered saline containing 0.15 mM NaCl and 0.1 % Tween-20 (Sigma, Madrid, Spain), for 90 min. The following antisera were used as primary antibodies: (1) anti-P15 (rabbit polyclonal antibody raised against a unique sequence of 15 aminoacids contained in SCO-spondin sequence [28]; 1:800 dilution); (2) anti-human SCO (rat polyclonal antibody raised against a protein extract of human fetal SCO [28]; 1:800 dilution); (3) anti-L1-CAM (monoclonal antibody kindly provided by Dr. Vance Lemmon [55]; 1:800 dilution); (4) anti-aquaporin 4 (rabbit polyclonal antibody; Sigma-Aldrich, USA;1:1500 dilution). Anti-rat or rabbit IgG conjugated to horseradish peroxidase (Pierce, Rockford, Ill., USA) was used at a 1:80,000 dilution, for 1.5 h. Incubations were carried out at room temperature in the dark. Immunoreactive polypeptides were detected using an enhanced chemiluminescence (ECL) system (Super Signal; Pierce) as instructed by the manufacturer. Molecular weight standards in the range of 10–250 kDa were used (Bio-Rad, Hercules, Calif., USA). Control blots were processed as above, with no incubation in the primary antibody.

## Abbreviations

CSF: cerebrospinal fluid
VZ: ventricular zone
NSC: neural stem cells
US: ultrasound
MRI: magnetic resonance imaging
CT: computerized axial tomography
EEG: electroencephalography
SCO: subcommissural organ
SA: sylvius aqueduct
TEPSI: psychomotor development test
WISC-R: wechsler intelligence scale for children
BVMG: bender visual motor gestalt test
